# A Novel Low-Cost Open-Hardware Platform for Monitoring Soil Water Content and Multiple Soil-Air-Vegetation Parameters

**DOI:** 10.3390/s141019639

**Published:** 2014-10-21

**Authors:** Giovanni Bitella, Roberta Rossi, Rocco Bochicchio, Michele Perniola, Mariana Amato

**Affiliations:** 1 School of Agriculture, Forestry, Food and Environmental Sciences, University of Basilicata, Potenza 85100, Italy; E-Mails: gianfranco.bitella@gmail.com (G.B.); bochicchiorocco@gmail.com (R.B.); michele.perniola@unibas.it (M.P.); mariana.amato@unibas.it (M.A.); 2 Consiglio per la Ricerca e la sperimentazione in Agricoltura—Research Unit for Cropping Systems in Dry Environments (CRA-SCA), Bari 70125, Italy

**Keywords:** Arduino, multi-sensor platform, open-hardware, microcontroller, soil water content, contactless temperature sensor, infrared thermometry, wireless data

## Abstract

Monitoring soil water content at high spatio-temporal resolution and coupled to other sensor data is crucial for applications oriented towards water sustainability in agriculture, such as precision irrigation or phenotyping root traits for drought tolerance. The cost of instrumentation, however, limits measurement frequency and number of sensors. The objective of this work was to design a low cost “open hardware” platform for multi-sensor measurements including water content at different depths, air and soil temperatures. The system is based on an open-source ARDUINO microcontroller-board, programmed in a simple integrated development environment (IDE). Low cost high-frequency dielectric probes were used in the platform and lab tested on three non-saline soils (ECe1: 2.5 < 0.1 mS/cm). Empirical calibration curves were subjected to cross-validation (leave-one-out method), and normalized root mean square error (NRMSE) were respectively 0.09 for the overall model, 0.09 for the sandy soil, 0.07 for the clay loam and 0.08 for the sandy loam. The overall model (pooled soil data) fitted the data very well (R^2^ = 0.89) showing a high stability, being able to generate very similar RMSEs during training and validation (RMSE_training_ = 2.63; RMSE_validation_ = 2.61). Data recorded on the card were automatically sent to a remote server allowing repeated field-data quality checks. This work provides a framework for the replication and upgrading of a customized low cost platform, consistent with the open source approach whereby sharing information on equipment design and software facilitates the adoption and continuous improvement of existing technologies.

## 1. Introduction

The need to increase water sustainability in agriculture has led to investments in precision irrigation techniques as well as a growing interest in phenotyping root traits for drought tolerance. Precision irrigation scheduling is based on actual site-specific real-time crop water requirements or measurements which can be logged and used to trigger irrigation when a threshold soil water content is reached [1,2]. Optimal sensors number and distances is crucial to obtain representative estimates of soil water availability at field-scale. Most soil hydraulic properties exhibit spatial correlation structures with ranges between 10 and 20 m [3] therefore a high number of sensors would be required to reflect this fine scale of variability. Phenotyping of root traits is an emergent, fast-growing field [4] and in particular phenotyping for the selection of drought-resilient genotypes [5] is receiving considerable attention as inferred by citation data increasing by 40% per year since the interest started to appear [6] and by the large investments in phenotyping [7]. Hi-tech phenotyping facilities are only available to a limited number of researchers, and do not satisfactorily address field-related issues. In fact field phenotyping is considered the real bottle-neck of effective variety selection [8].

Monitoring changes in soil water content at different depths through the rhizosphere gives key information on plant water-uptake rates and strategies but also on parameters such as rooting depth (RD) which can be estimated indirectly by soil-water depletion curves in the profile during a water-supply-limited period [9]. Soil can be considered “a dynamic fresh-water reservoir” [10] with a spatially variable water holding capacity. Therefore strategies for increasing efficiency in water use require a high spatial coverage and time-dynamics of soil water content information.

Soil water content can be either measured directly (*i.e.*, by gravimetric method) or indirectly by non-destructive (highly repeatable) measures of another parameter (*i.e.*, soil permittivity, temperature, resistivity) which is affected by soil water content change, this surrogate data variability is then converted to water content using either physically-based laws or empirical calibration curves. Soil water content sensors, including neutron probes and large scale methods such as ground penetrating radar, have been reviewed by Bittelli [11], the most used equipment for water monitoring at local scale are the dielectric sensors which measure soil dielectric permittivity using capacitance or time domain technologies (TDR): These sensors buried at different depths and at multiple locations provide useful soil water content time series. While TDR has long been the best established technology [12], capacitance probes are increasingly adopted for their simplicity of use, the low cost [13] and their sensitivity to changes in soil permittivity comparable to that of the TDR [14–16]. The main drawback of dielectric sensors is their sensitivity to soil electrical conductivity and temperature, which often requires specific calibration/correction procedures [17–19]. The simultaneous availability of other data, such as soil temperature would be therefore necessary in some cases (e.g., for correction of temperature-dependent capacitive probe readings) and desirable in many contexts such as root research. Soil temperature not only affects root growth development and turnover but, together with water availability, also root architectural traits such as root gravitropic angle [20], which is an important trait linked to water acquisition efficiency [21–23]. Moreover low root zone temperature can worsen the effects of other abiotic and biotic stresses such as salinity [24] and pathogen infections rate [25]. Temperature data in the root zone are therefore relevant to improve root data interpretation relative to other sources of variation or when temperature becomes itself an experimental factor. Soil temperature data are especially needed in contexts where decupling between air and soil temperature is expected, such as under greenhouse horticulture [24]. Coupling soil/atmosphere data with plant-based measurements such as canopy temperature (CT) can be used for non-destructive high throughput assessment of plant water stress and drought avoidance strategies [26,27]. Crop water status can be indirectly assessed by measuring canopy temperature and its reduction relative to air temperature. The cooling effect of transpiration makes canopy temperature inversely proportional to stomatal conductance, leaf water potential and turgor [28], these authors found CT negatively correlated to rice grain yield in drought stressed plants. CT was also used to develop crop-temperature-based water stress indices [29,30]. CT can either be measured through thermocouples or remotely through infrared thermometry (IRT), which measures the long-wave infrared radiation emitted by an object that is a function its temperature. IRT measurements have advantages over other methodologies for the assessment of plant water status, such as leaf water potential measurements by leaf chamber, which is relatively slow and therefore unsuitable to screen large breeding nurseries [26]. It is also faster than contact-based probes and is suited for on-the-go measurements [31]. For the mentioned reasons IRT has been widely used in agronomic research providing a reliable, simple, and fast tool for selecting cultivars with greater dehydration avoidance mechanisms [26,27,32] as well as for irrigation scheduling [33]. There are several high quality industrial devices available on the market however their high cost and complexity has limited their adoption in agricultural production and has progressively led to the development of alternative low-cost instruments [34,35]. Mahan and Yeater [34] compared the performances of some high quality industrial and low-cost IRT sensors in agricultural settings and they proved that the measurements from the two categories were similar over a 13–35 °C range, which is the range experienced by crops in most temperate regions, hence low-cost devices might be a sound alternative in many agricultural applications. In any agricultural field experiment, independently from the specific target, air temperature and humidity are the most frequently measured environmental parameters since they determine evapotranspiration demand, and control crop phenology and growth. Such measurements complement agronomy trials giving information on environmental effects superimposing experimental factors. The necessity of spatially distributed weather measurements, especially desirable in complex terrains, gave impulse to the development of new generation low-cost sensors increasingly used to obtain high spatial coverage [36]. Multi-sensor devices are increasingly needed, along with the relevant technologies to store, retrieve and remotely transmit a large and widespread amount of data. To this end, when a large number of measurements sites (*nodes*) are involved the use of Wireless Sensor Networks (WSN) is becoming a “dominant option” [37]. Wireless transmission technologies reviewed by Ruiz-Garcia and co-authors [38] include Zigbee and Bluetooth protocols and modem-based connections such as GPRS and GSM, often coupled with internet applications [39].

The cost of data loggers, multiplexers and sensors, however, limits the number of sensors and the measurements frequency. Recent advances in the field of “open hardware” components open new alleys for the development of measurement stations that can be connected with wireless transmission devices in order to fill a gap in low-cost tools for high spatio-temporal coverage of soil water data and multi-sensor acquisition. “Open hardware” philosophy in analogy with the concept of “open source software” aims at providing free and transparent access to hardware design, projects and code so that users can easily share, customize and upgrade their systems [40,41].

The objective of this work was the design of a low cost “open hardware” platform for the measurement, recording and wireless transmission of data on water content at different depths, air, soil and canopy temperatures.

The system is based on an open source microcontroller board of the series Arduino [42], handling both analog and digital sensors. Arduino platforms are the most famous and fast spreading electronic prototype development, and they have already been used in research for a variety of applications [43–47]. The circuit board, all the electronic components and software to program the microcontroller are freely available. Ease of use/programming is matched with the affordable prices of the electronic components, thus belonging to the group of so-called democratizing technology [48]. The platform is equipped with low-cost, new generation, dielectric sensors now available on the market, and this reduces the cost of data acquisition even further. Measurements at several sites and depths therefore become affordable, and this allows the space-time density of data required for applications aimed at water saving.

## 2. Experimental Section

### 2.1. Platform

**Figure 1.**
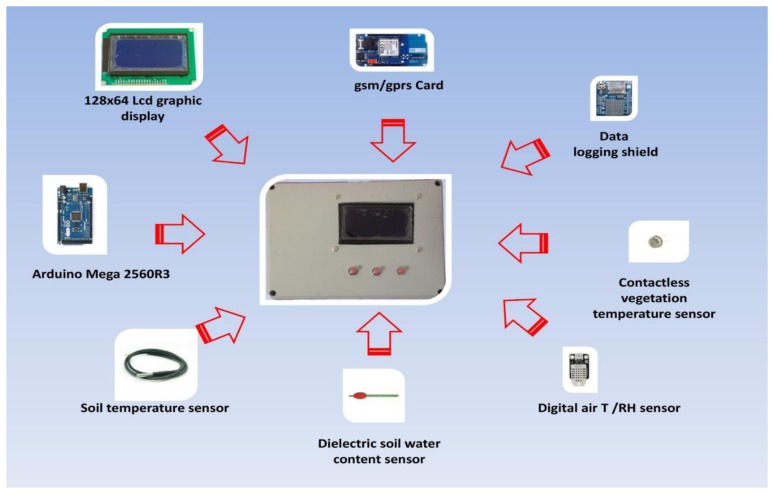
Components of platform.

**Figure 2.**
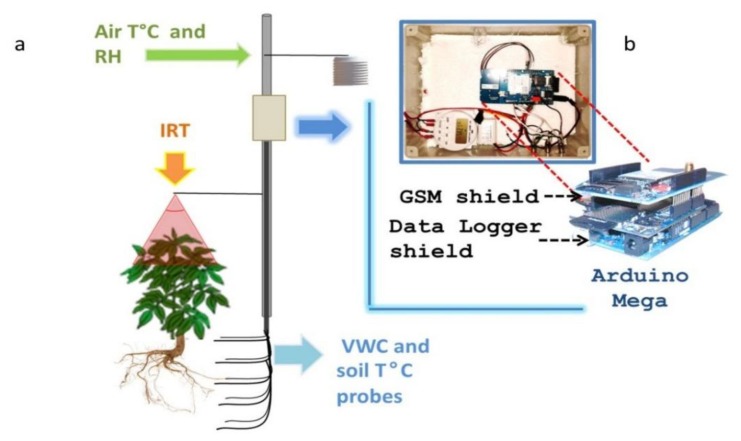
Platform architecture: (**a**) schematic representation of a measurement unit; (**b**) (inset) electronic components inside the indoor enclosure (top-view) and magnification of the micro-controller board and the superimposable expansion shields.

**Scheme 1.**
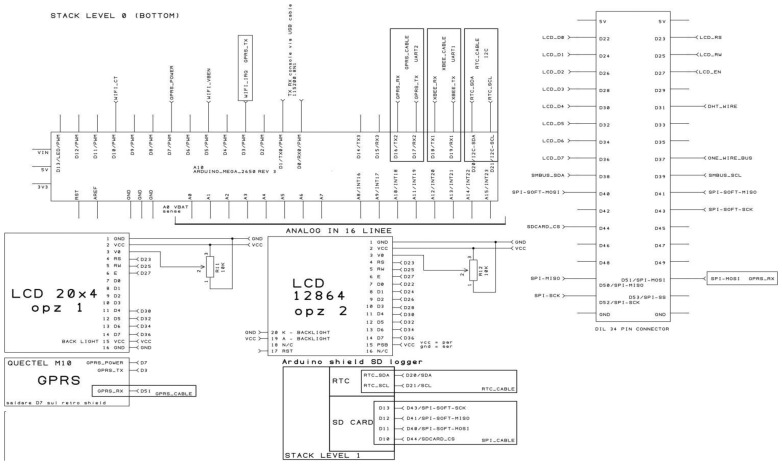
Schematic electric diagram of the platform.

**Scheme 2.**
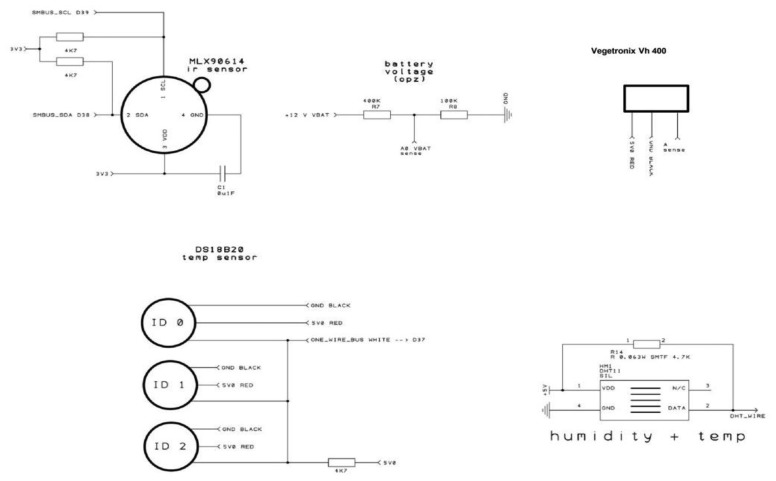
Schematic electric diagram of sensors.

The prototype platform we developed features several components (Figure 1) organized in 3 units depicted in schemes I and II:

- Microcontroller board;
- Expansion boards:
  (a) Data logging shield
  (b) GPRS card for wireless transmission
  (c) Optional LCD display for real-time data displaying;
- Sensors:
  Platform components are shown in Figure 1, the system architecture is depicted in both Figure 2 (schematic representation of a measurement unit) and the schematic electric diagrams (Schemes 1 and 2). Schemes 1 and 2 are the electrical schematic diagrams of the circuit board. The electric diagram is an abstract representation that shows how all the platform components (sensors, data-loggers and micro-controller) are connected between themselves through input and output pins and where all the auxiliary electronic components (such as resistors and capacitors) are placed. These schemes can be used as a reference to reproduce the system.

#### 2.1.1. The Microcontroller

The microcontroller is an open source board of the Arduino series [42], handling both analogic and digital sensors, and simply connectable to a computer through a USB cable. The Arduino microcontroller can be programmed through the multi-platform development environment (IDE). All Arduino boards are designed to allow easy connection with other expansion boards (named shields) through a superimposable sequence system. The board used for this project is Arduino Mega 2560 Rev.3.0 featuring an ATMEL microcontroller (ATmega2560) and equipped with 54 digital inputs/outputs and 16 analog inputs (Figure 1).

For field and lab measurements technical specifications of the platform to be constructed around the Arduino microcontroller include:

- Weather proof electrical cabinet IP65 of at least 36 cm × 25 cm × 14 cm for field applications.
- Indoor plastic enclosure to protect electronic components of at least 24 cm × 19 cm × 9 cm for field and lab applications.
- Protection of cables from animal damage through plastic pipes of 4–5 cm diameter.
- External stabilized 12 V–5 V DC/DC power converter.
- Power converter of at least 2 A.
- Capability of data storage of at least 1 GB.
- Capability for data remote transmission with the GPRS protocol at a speed of at least 85.6 kbps.
- In order to ensure flexibility and modularity of sensors it must be capable of supporting the connection to all of the analog ports which means at least 16 water content probes, 5 temperature probes, 1 IR sensor and 1 weather station.

Technical specifications of sensors include:

- Weather sensors needed to operate in the temperature range of −10 to +60 °C and relative humidity from 10% to 90%
- Temperature sensors needed to measure temperatures at least in the range −15 to 60 °C, Humidity sensors needed to Measure relative humidity from 10% to 90%
- Soil water sensors needed to measure soil water content in the range 5% to 50%
- IR sensors need to measure a range from 10 to 40 °C.

Furthermore, for compatibility with the Arduino Mega, sensor signal output must range between 5 mV and 5 V.

#### 2.1.2. The Expansion Boards

##### (a) Data Logging Shield

The data acquired from the sensors are recorded on a non-volatile and preformatted fat 16–32 memory card type secure digital (SD) through the fully integrated expansion card produced by Adafruit [49]. Data acquisition and timing is completely programmable. The card supports memory cards up to 2 GB and include a real-time clock (RTC) chip ds 1307 [50], it was battery powered independently from the Arduino board. The RTC allows keeping of accurate time, even in the temporary absence of the main power, which is necessary for a correct combination of the time information to the data from the sensor.

##### (b) GPRS Card for Wireless Transmission

In order to remotely monitor the platform, the system is equipped with a transmission GPRS card connected to the microcontroller through two analog pins at a speed of 115.2 kbps, and able to exchange data remotely to the theoretical maximum speed of 85.6 kbps using a sim phone card. We used the official Arduino GPRS shield developed with Telefonica [51] equipped with a radio modem M10 by Quectel [52] quad-band GSM/GPRS that works at frequencies of GSM850MHz, GSM900MHz, DCS1800MHz and PCS1900MHz. It supports TCP/UDP and HTTP protocols through a GPRS connection. Data recorded in txt/xml format on the SD (see Section 2.1.2 (a)) are transferred to a remote server (see Section 2.1.4) on a daily basis for easy access and to allow periodic monitoring of the proper functioning of the acquisition system.

##### (c) The Graphic Display

The graphic display used is the mod. 12,864 ZW commercialized by www.robot-domestici.it [53]. It is a graphical liquid crystal display (LCD) 64 × 128 with LED backlight. The display is connected to the microcontroller by a parallel mode connection. This connection required 8 digital pin and 5 V power.

#### 2.1.3. Sensors

The sensors used for monitoring water status are dielectric probes operating at 80 MHz [54] and require a power supply with a voltage between 3.3 and 20 V DC. They provide a voltage reading between 0 and 3 V as a function of the water content of the medium surrounding the sensor with an average consumption of less than 7 mA. Besides the low cost (Table 2), the probes offer several advantages: As reported by the manufacturer [54] and shown by experimental results [55] the sensors are insensitive to water salinity, the probe is small and rugged and does not corrode over time as conductivity-based probes usually do. Communication with the data-logger is analog and occurs through the analog to digital converter at 10-bit resolution, which is included in the Arduino Mega board and converts voltage measurements in the range 0–1023 V. When a voltage of 5 V is provided to the probes, a resolution of about 0.005 V (5/1023) is obtained [45]. Temperature and humidity sensors were selected among those with a digital interface in order to leave the largest possible number of free analog ports on the card to allow for the additional installation of a great number of soil water content sensors. The soil temperature sensor is a Dallas DS18B20 digital thermometer [56] capable of detecting temperatures between −55 and 125 °C with an accuracy of ±0.5 °C from −10 °C to +85 °C. In our system all of the sensors (except the IR probe that required 3 V) were connected to a stabilized 12–5 V DC/DC converter with a power of 3 A that was sufficient to supply the maximum number of sensors connectable to the micro-controller. The thermometer, incorporated in a protective stainless steel capsule, was connected to the microcontroller using a 4.7 pull up resistor (see Scheme II) and a 3.5 mm stainless jack stereo connection. The air temperature and humidity sensor is a DHT22, commercialized by Exp-tech.Inc [57], it allows a simultaneous measurement of temperature and relative humidity (RH) with a range between −40 and 125 °C and an accuracy of ±0.5 °C for temperature and between 0% and 100% RH with an accuracy of 2%–5%. The temperature of vegetation was acquired by a small size non-contact infrared thermometer MLX90614ESF-BAA-00TU-ND that is marketed factory-calibrated by Melexis Microelectronic Systems [58]. This sensor is able to measure object temperature between −70 °C and +380 °C with a resolution of 0.01 °C and an accuracy of 0.5 °C. The field of view is 90. The sensor, encapsulated in a plastic cable gland, was fixed to an adjustable angle support in order to manipulate distance and position to optimize the target view. The interface used for the connection with the microcontroller board was I^2^C, which is a type of serial communication.

Prices of platform components and sensors are reported in Tables 1 and 2 respectively, shipping costs were not included. For field applications other additional consumable materials might be needed such as plastic pipes, metal poles, cable glands, weather proof electronic cabinet and clamps. These costs (amounting all together less than 100 € on average) were not reported in details since different materials could be purchased depending on size requirements and expected deployment time.

#### 2.1.4. Data Management

Data management of the platform involves the acquisition, storage and access/transmission of temporal or spatio-temporal data of different categories. The measurement system we propose involves multiple soil water content and soil temperature sensors and therefore a spatio-temporal array of water content and soil temperature data and a temporal dataset for canopy temperature, air temperature and humidity (one sensor each), for each measurement stations. The number and type of sensors to be read as well as sensors acquisition pace is programmable. Data from all sensors are acquired simultaneously.

Data are stored in the data logger to a maximum capacity of 2GB in the txt/xml format. There are three options for access to stored data:

(1) Periodical data retrieval from the data-logger by direct access to the SD card.
(2) Remote access to data sent to a server through the GPRS transmission. This allows managing high data throughput as described in Section 2.1.2 (b). The desired pace of data transmission, though, is set through Arduino code, in our example we set it to once a day in order to save energy and traffic fares.
(3) Real-time access through the LCD display for quick checks, especially in case of problems with the transmission protocol.

All three are available with the platform as described, but options 2 and 3 may be excluded if a display or a transmission unit is not desired. Since there are many possible causes of failure of data retrieval systems, the use of multiple options, though, is strongly advised in order to reduce the risk of data loss or temporary lack of accessibility.

Also, data format allows integrating the platform with existing applications for data visualization, storage and processing on database servers.

This data management architecture allows acquiring and transmitting/accessing information from multiple measurement points throughout field or lab settings, and to use information after processing or as such for real-time decisions such as those related to irrigation events when thresholds are reached in one or multiple sites.

### 2.2. The Platform Power Supply

To power the platform 7–12 V direct current (DC) is recommended. For testing we used an electronic adapter AC 110–220 V to DC 12 V 3 A connected to the electric grid. For open field application, power supply can be provided through a common lead-acid battery to 12 V and the environmental sensors are powered directly by a 12–5 V 3 A high-efficiency digital switch, bypassing the on board linear converter. The battery state-of-charge is monitored simultaneously with the environmental sensors, using a voltage divider, depicted in the schematic diagram of the system. The voltage divider, connected directly to the battery (12 V), produces an output voltage (2.4 V) readable by the Arduino board through its analogical ports.

The overall electronics are protected in a weatherproof case and receive signals from the environmental sensors through connections with quick mounting of the 3.5 mm stereo jack type fixed on the wall of the casing.

The software code is freely available directly from the authors on request.

### 2.3. Soil Water Content Sensors Calibration

The platform was calibrated for the detection of soil water content in non-saline soils (ECe1: 2.5 < 0.1 mS/cm) with three different types of grain size characteristics: a sandy soil (sand 76.48%, silt 16.82%, clay 6.7%), a silty clay soil (sand 30.66%, silt 45.06%, clay 24.28%), and a sand. Each soil was air-dried and sieved to 2 mm, and used to fill 300 mL glass containers. Samples of different water content were obtained by adding different amounts of tap water at the temperature of 20 °C, covering the containers with a screw-lid and equilibrating at 20 °C for 24 h. Containers were then opened and water content was measured with VH400 rev.6.0 probes (Vegetronix™). All samples were weighted fresh and after oven drying at 105 °C for gravimetric water content determination and converted to volumetric water content by multiplying for the bulk density. Stability of dielectric probe readings to temperature was tested by keeping the probes fully immersed in deionized water and recording probe output sensitivity to temperature changes from 3 °C to 50 °C.

### 2.4. Statistical Analysis

The relationship between volumetric water content (VWC) and sensor reading was non-linear following a sigmoidal growth. Data were modelled using a three parameters logistic model in the form:

(1) $$y=\frac{\text{asym}}{1+e^{\frac{\left(xmid-x\right)}{\text{scal}}}}$$

with: y = volumetric water content (Kg·m^−3^); x = sensor output reading (V).

The model was fitted to the pooled data set (*overall model*) and then to each soil texture (*single texture models*). Parameters significance and goodness of fit statistics were calculated. In order to test if soil-specific calibration curves were required, significant differences between single-texture models parameters were assessed using the likelihood-ratio test. Models were validated by a leave-one-out cross validation (LOOC). All data analysis was accomplished in the “R software environment for statistics” [59].

## 3. Results and Discussion

### Output Data and Calibration

**Figure 3.**
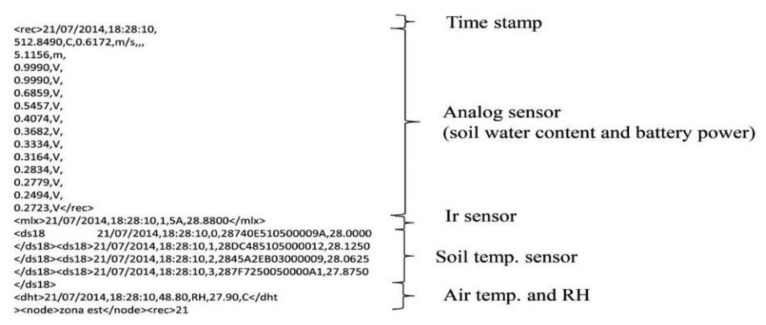
Example of log file.

An example of the log file with data collected from the fully-equipped platform is shown in Figure 3.

**Figure 4.**
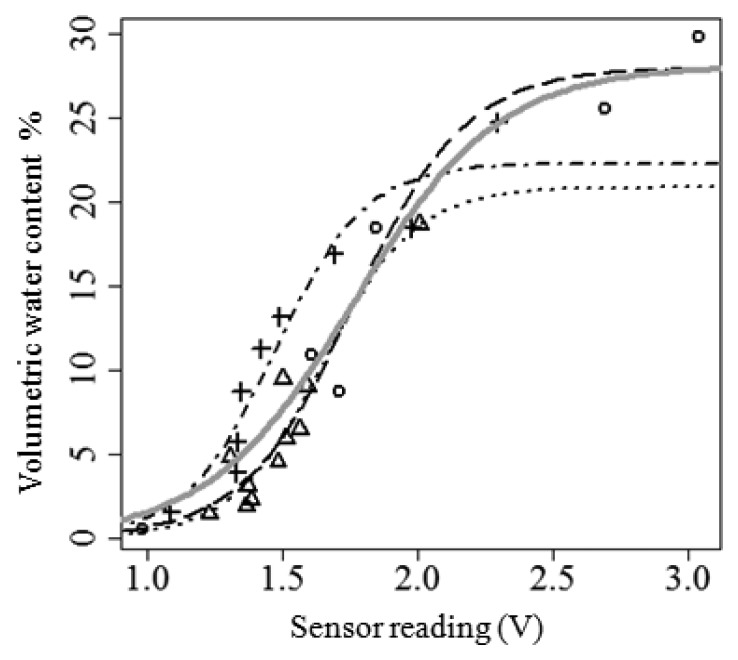
Calibration of soil water content dielectric probes on three soil media. Symbols = data points: Open triangles = sand; open circles = loamy-clay soil; crosses = sandy soil. Lines = model results: Dotted line = sand; dashed line = loamy clay soil; dotted-dashed line = sandy soil; solid grey line = overall model.

Output from the analog soil water content sensor in Volts *versus* direct measurements used for calibration are reported in Figure 4 and statistics of the calibration models are shown in Table 3.

Following data behaviour the relationship between VWC and sensor reading was modelled by a 3-parameters logistic function. The logistic or autocatalytic model is a nonlinear function defined by three parameters referred to as asymptote, xmid and scal in Table 3 (models summary). The asymptote is the final limiting value attained by the response variable (VWC), xmid is the input value (sensor output reading (V)) at which the inflection point is reached and the “scal” is the inherent growth rate. The purpose of this empirical model was to provide a framework to convert sensor output to water content values, In Table 3, for each texture and for the pooled data (overall model), parameters estimates are reported together with their standard error and significance. The table shows that regardless of the difference in soil texture, the relationship between sensor reading and soil water content is non-linear and always follows a sigmoid behaviour. In both pooled and single-texture models the parameters are always significant (*p*-value < 0.05) with the sole exception of the scale parameter in the loamy-clay model. In our data the sandy-loam model shows poor predictions at extremes values (water content below 5% and above 20%). The sand model showed scattered values between 5% and 10% VWC). This might be due to the low cohesion of sand at low water content that causes particles displacement and the possible creation of voids at the interface with the probe. This might be due to the low cohesion of sand at low water content that causes particles displacement and the possible creation of voids at the interface with the probe. Geesing and co-authors [60] discuss the effects of bulk density and particle displacement on capacitive probe readings, highlighting the importance of developing distinct calibration curves for coarse and fine textured soils. In our analysis all of the single-texture model parameters show very similar values, the loamy-clay soil has the highest asymptote and the slowest “growth rate” compared to the other textures. Nevertheless, the single-texture models were found not significantly different (*p*-values > 0.05) at the likelihood ratio test [61] therefore, as is also suggested by manufacturer, at least for the ranges of texture and water content that we tested soil-specific calibration equations are not required.

The overall model fits the data very well (R^2^ = 0.89) showing a high stability, being able of generating very similar RMSEs during training and validation (RMSE_training_ = 2.63; RMSE_validation_ = 2.61).

Despite advantages of dielectric‐based capacitance probe (CP) leading soil-monitoring sensors, these methods are prone to temperature and salinity drifts. Analytical models for salinity correction and temperature dependent scaled voltage techniques have been validated for different soil types [62,19]. Laboratory calibration of commercial dielectric soil water sensors have shown both linear and non-linear responses to increasing water content depending on soil type, therefore soil-specific calibration equations have been recommended [62]. In field application a calibration method based on the information-sharing in wireless sensor network incorporated spatially variable temperature and salinity data [63].

**Figure 5.**
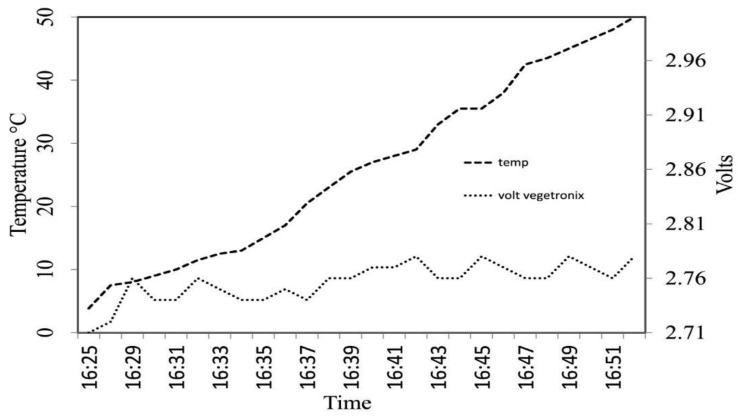
Variation of temperature and dielectric probe readings in water during the temperature-drift experiment.

Figure 5 reports the variation of temperature and the soil water content probe readings during the temperature-drift experiment in water. A difference of about 0.07 V between readings at 3 and 50 °C was found, with a significant drift of 0.0009 V·°C^−1^ (y = 0.0009x + 2.733; R^2^ = 0.54) consistent with previous findings [62].

Mcmichael and Lascano [62] tested the temperature drift of dielectric probes (Model EC20 probe, Decagon Devices, Pullman, WA) at different water content, and found that sensitivity to temperature increases with volumetric water content, ranging from 0.5 mV·°C^−1^ in dry soil to 5.5·mV·°C^−1^ at 0.20 Kg·m^−3^ VWC. This indicates that temperature effects on capacitive probes cannot be ignored especially in the wetter range, and hence temperature data would always be required for correction. Since in field calibration changes in bulk density as well as large temperature fluctuations can be expected, the authors [62] suggest to reduce artifacts linked to temperature sensitivity of CP, place the probes beneath the first soil layers thus avoiding steep gradients of both temperature and water content. In our case the Vegetronix VH400 probe proved to be sensitive to temperature changes in wet conditions (in water) thus temperature data would always be required. A positive linear trend exists. The determination coefficient though is relatively small, the observations are scattered around the regression line and the range of the response variable is narrow (between 2.71 and 2.78 V). The lack of fit of the linear model is partially explained by the fact that between 30 and 50 °C the largest fluctuation of the response variable occur. Within this interval the regression line levels off. Indeed, when the full range of data was modeled by a power relationship (data not shown), which approaches a plateau with increasing temperatures, a better fit is achieved; the determination coefficient rises to 0.65. When the dataset were split in two subsets (from 3 to 30 °C and from 30 to 50 °C) and each one was modeled by a linear regression for the first subset the linear trend was significant (y = 2.7209 + 0.0017x; R^2^ = 0.60 *p*-value < 0.001) while the dependency disappeared at large values of temperature. For temperature corrections the linear model fit up to values of 30 °C could be used at least below the soil surface, since in most agricultural settings, this temperature threshold value would be plausible in field soils with a water content near saturation (wetter range). The fluctuations of sensor readings as well as the non-linear sensitivity to temperature at high water content are consistent with other studies [18,62], regarding sensor output fluctuations a feasible option to reduce data noise during calibration would be increasing the sensor sampling frequency and averaging out multiple measurements [64]. To develop an absolute temperature correction procedure, however, temperature drift in soil remains to be tested along with possible hysteresis phenomena [62]. Albeit this preliminary fast calibration procedure confirmed the need of equipping the platform with a number of soil temperature sensors conforming to the number of water content probes. Kizito and co-authors [18] demonstrated that increasing dielectric probes measurement frequency up to 70 MHz considerably reduces the sensitivity to conductivity and texture, while sensitivity to temperature was unaltered by the frequency but was found consistently low across soil/solutions and conductivity ranges. The authors argued that even if temperature effects are correctable, the temperature dampening effect in soil would probably reduce this need in many applications. Our probe operates with a higher frequency of measurements (80 MHz) and this makes it even less sensitive to texture and salinity effects while temperature drift, if necessary, can be easily corrected by data processing. The sensor is sensitive to even small changes in water content. This sensitivity is very useful in predicting changes in matric potentials and its implications in variations of soil strength and plant available water.

For this prototype we chose data transmission via GPRS to allow automated data retrieval and remote control of sensors functioning and battery state-of-charge even from remote locations where Wi-Fi is not available. Nevertheless this system can be easily upgraded by adding a low-power Wi-Fi expansion board (*i.e.*, xbee transmission [65]) that allows wirelessly transmission and multipoint communications [46]. For this platform some of the selected sensors are inexpensive to the point of being considered disposable, this is advantageous when probes are buried at considerable depths and thus cannot easily be retrieved. In the case of malfunctions, all sensors can be easily replaced in the field with the aid of a screwdriver. The choice of sensor stems from of a combination of factors such as the objective of measurements, sensor accuracy within the relevant range of measurable values, ease of use and cost. Opting for low-cost equipment, as argued by Mahan and Yeater [34] is not necessarily aimed at reducing the cost of measurements itself but rather on having, at the same cost of a high quality sensor, an increased data density and thus better encompass the spatio-temporal variability of crop attributes and processes.

## 4. Conclusions

An Arduino microcontroller board provided the basis for a multi-sensor platform for the measurement and remote transmission of soil water content alone or coupled with other relevant soil, vegetation and atmosphere parameters. High-frequency dielectric sensors mounted on board proved to be subjected to temperature drifts; hence data collection must be integrated by soil temperature measurements. Meanwhile, the probes are sensitive to even small changes in water content and a single calibration equation can be used, at least in sandy, sandy-loam and loamy-clayey soils.

This work provides a framework for the replication and upgrading of a customized low cost platform, consistent with the open source approach whereby sharing information on equipment design and software facilitates the adoption and continuous improvement of existing technologies. The low cost of all components allows the envisaging of applications where a high number of sensors are required and/or sensors need to be placed in locations where they cannot be easily recovered.

## Figures and Tables

**Table 1.** Prices of the main components of the platform (source [53]).

Platform Electronic Components	Price in €, Vat Excluded
Adafruit datalogging shield	20
Arduino mega	39
Arduino GSM Shield	69
Optional display	18
DC/DC converter 12 V to 5 V 3 A	5
SD card 2 GB	2
AC 110B-220 V to DC 12 V 3 A regulated transformer power supply	10
Additional electronic materials (connectors, resistors, capacitors)	20
Electronic enclosure	10
Total	193

**Table 2.** Prices for each sensor (source [53,54]), type of measurement and possible applications.

**Sensors**	**Type of Measurement**	**Applications**	**Price in € (Vat Excluded)**
Vegetronix VH400	Soil water content	hydrology, agronomy, soil physics, irrigation, plant physiology, phenotyping, root ecology	28.2
DS18B20	Soil temperature	agronomy, soil physics, irrigation, plant physiology, phenotyping, shoot and root growth and development	7.24
MLX90614	Air temperature and Relative Humidity	agronomy, irrigation, plant physiology, meteorology, crop water requirements	9.80
melexis IR Sensor	Canopy temperature	agronomy, irrigation, plant physiology, phenotyping, crop ecology, plant water stress response	19.95

**Table 3.** Summary statistics of calibration models for soil water content sensors. For each soil texture and for the overall model the logistic function parameters estimates were reported together with their standard error and significance, normalized root mean square error (NRMSE) as a measure of goodness of fit. The asterisks “*”indicate the coefficients significance levels. The symbols are displayed as follows: Any *p*-value < 0.001 was designated with three (***) asterisks; *p*-values >= 0.001 and <= 0.01 are shown with two (**) asterisks, *p*-values > 0.01 and < 0.05 are shown with one (*) asterisk.

**Texture**	**Parameters**	**Estimate**	**Std. Error**	**t-Value**	**Pr(>|t|)**		**NRMSE**
Sandy-Loam	xmid	1.470	0.054	27.242	1.62E-07 ***	Residual standard error: 2.307 on 6 d.f. Number of iterations to convergence: 16 Achieved convergence tolerance: 6.816e-06	0.081
	scal	0.166	0.052	3.199	1.86E-02 *		
	Asym	22.263	2.032	10.954	3.44E-05 ***		

Loamy-Clay	xmid	1.765	0.069	25.500	1.32E-04 ***	Residual standard error: 2.951 on 3 d.f. Number of iterations to convergence: 8 Achieved convergence tolerance: 3.206e-06	0.071
	scal	0.206	0.110	1.870	1.58E-01		
	Asym	27.932	2.233	12.510	1.10E-03 **		

Sand	xmid	1.650	0.096	17.172	1.35E-07 ***	Residual standard error: 1.734 on 8 d.f. Number of iterations to convergence: 5 Achieved convergence tolerance: 2.201e-07	0.086
	scal	0.168	0.057	2.969	1.79E-02 *		
	Asym	20.840	3.775	5.520	5.60E-04 ***		

Overall	xmid	1.762	0.065	26.980	2.00E-16 ***	Residual standard error: 2.802 on 23 d.f. Number of iterations to convergence: 4 Achieved convergence tolerance: 2.366e-06	0.09
	scal	0.268	0.044	6.070	3.43E-06 ***		
	Asym	28.034	2.146	13.070	3.97E-12 ***		

## References

[1] Hedley C.B., Yule I.J. (2009). Soil water status mapping and two variable-rate irrigation scenarios. Precis. Agric..

[2] Goumopoulos C., O'Flynn B., Kameas A. (2014). Automated zone-specific irrigation with wireless sensor/actuator network and adaptable decision support. Comput. Electron. Agric..

[3] Wendroth O., Koszinski S., Pena-Yewtukhiv E. (2006). Spatial Association among Soil Hydraulic Properties, Soil Texture, and Geoelectrical Resistivity. Vadose Zone J..

[4] Wasson A.P., Richards R.A., Chatrath R., Misra S.C., Prasad S.V., Rebetzke G.J., Kirkegaard J.A., Christopher J., Watt M. (2012). Traits and selection strategies to improve root systems and water uptake in water-limited wheat crops. J. Exp. Bot..

[5] Tuberosa R. (2012). Phenotyping for drought tolerance of crops in the genomics era. Front. Physiol..

[6] Passioura J.B. (2012). Phenotyping for drought tolerance in grain crops: When is it useful to breeders?. Funct. Plant Biol..

[7] International Plant Phenotyping Network-IPPN. http://www.plant-phenotyping.org.

[8] Araus J.L., Cairns J.E. (2014). Field high-throughput phenotyping: The new crop breeding frontier. Trends Plant Sci..

[9] Dardanelli J.L., Bachmeier O.A., Sereno R., Gil R. (1997). Rooting depth and soil water extraction patterns of different crops in a silty loam Haplustoll. Field Crop. Res..

[10] Hedley C.B.B., Roudier P., Yule I.J.J., Ekanayake J., Bradbury S. (2013). Soil water status and water table depth modelling using electromagnetic surveys for precision irrigation scheduling. Geoderma.

[11] Bittelli M. (2011). Measuring soil water content: A review. HortTechnology.

[12] Topp G.C., Davis J.L. (1985). Time-Domain Reflectometry (TDR) and Its Application to Irrigation Scheduling. Adv. Irrig..

[13] Fares A., Polyakov V. (2006). Advances in Crop Water Management Using Capacitive Water Sensors. Adv. Agron..

[14] Gardner C.M.K., Dean T.J., Cooper J.D. (1998). Soil water content measurement with a high-frequency capacitance sensor. J. Agric. Eng..

[15] Robinson D.A., Gardner C.M.K., Cooper J.D. (1999). Measurement of relative permittivity in sandy soils using TDR, capacitance and theta probes: Comparison, including the effects of bulk soil electrical conductivity. J. Hydrol..

[16] Seyfried M.S., Murdock M.D. (2004). Measurement of Soil Water Content with a 50-MHz Soil Dielectric Sensor. Soil Sci. Soc. Am. J..

[17] Bittelli M., Salvatorelli F., Pisa P.R. (2008). Correction of TDR-based soil water content measurements in conductive soils. Geoderma.

[18] Kizito F., Campbell C.S., Campbell G.S., Cobos D.R., Teare B.L., Carter B., Hopmans J.W. (2008). Frequency, electrical conductivity and temperature analysis of a low-cost capacitance soil moisture sensor. J. Hydrol..

[19] Scudiero E., Berti A., Teatini P., Morari F. (2012). Simultaneous monitoring of soil water content and salinity with a low-cost capacitance-resistance probe. Sensors..

[20] Nakamoto T. (1995). Gravitropic Reaction of Primary Seminal Roots of Zea mays L. Influenced by Temperature and Soil Water Potential. J. Plant Res..

[21] Christopher J., Christopher M., Jennings R., Jones S., Fletcher S., Borrell A., Manschadi A.M., Jordan D., Mace E., Hammer G. (2013). QTL for root angle and number in a population developed from bread wheats (Triticum aestivum) with contrasting adaptation to water-limited environments. Theor. Appl. Genet..

[22] Sanguineti M.C., Li S., Maccaferri M., Corneti S., Rotondo F., Chiari T., Tuberosa R. (2007). Genetic dissection of seminal root architecture in elite durum wheat germplasm. Ann. Appl. Biol..

[23] Singh V., van Oosterom E.J., Jordan D.R., Hammer G.L. (2012). Genetic control of nodal root angle in sorghum and its implications on water extraction. Eur. J.Agron..

[24] HE Y., Yang J., Zhu B., Zhu Z. (2014). Low Root Zone Temperature Exacerbates the Ion Imbalance and Photosynthesis Inhibition and Induces Antioxidant Responses in Tomato Plants Under Salinity. J. Integr. Agric..

[25] Watt M., Silk W.K., Passioura J.B. (2006). Rates of root and organism growth, soil conditions, and temporal and spatial development of the rhizosphere. Ann. Bot..

[26] Blum A., Mayer J., Gozlan G. (1982). Infrared thermal sensing of plant canopies as a screening technique for dehydration avoidance in wheat. F. Crop. Res..

[27] Winterhalter L., Mistele B., Jampatong S., Schmidhalter U. (2011). High throughput phenotyping of canopy water mass and canopy temperature in well-watered and drought stressed tropical maize hybrids in the vegetative stage. Eur. J. Agron..

[28] Ndjiondjop M.N., Manneh B., Cissoko M., Drame N.K., Kakai R.G., Bocco R., Baimey H., Wopereis M. (2010). Drought resistance in an interspecific backcross population of rice (Oryza spp.) derived from the cross WAB56-104 (O. sativa) × CG14 (O. glaberrima). Plant Sci..

[29] Ingram K., Real J., Maguling M. (1990). Comparison of selection indices to screen lowland rice for drought resistance. Euphytica.

[30] Vijaya Kumar P., Ramakrishna Y., Ramana Rao B., Khandgonda I., Victor U., Srivastava N., Rao G.G.S. (1999). Assessment of plant-extractable soil water in castor beans (Ricinus communis L.) using infrared thermometry. Agric. Water Manag..

[31] Mahan J.R., Conaty W., Neilsen J., Payton P., Cox S.B. (2010). Field performance in agricultural settings of a wireless temperature monitoring system based on a low-cost infrared sensor. Comput. Electron. Agric..

[32] Guimarães C.M., Stone L.F., Lorieux M., de Oliveira J.P., de O'Alencar G.C., Dias R.A.A. (2010). Infrared thermometry for drought phenotyping of inter and intra specific upland rice lines. Rev. Bras. Eng. Agríc. Ambient.

[33] Sezen S.M., Yazar A., Daşgan Y., Yucel S., Akyıldız A., Tekin S., Akhoundnejad Y. (2014). Evaluation of crop water stress index (CWSI) for red pepper with drip and furrow irrigation under varying irrigation regimes. Agric. Water Manag..

[34] Mahan J.R., Yeater K.M. (2008). Agricultural applications of a low-cost infrared thermometer. Comput. Electron. Agric..

[35] O'Shaughnessy S.A., Hebel M.A., Evett S.R., Colaizzi P.D. (2011). Evaluation of a wireless infrared thermometer with a narrow field of view. Comput. Electron. Agric..

[36] Hubbart J., Link T., Campbell C., Cobos D. (2005). Evaluation of a low-cost temperature measurement system for environmental applications. Hydrol. Process..

[37] Garcia-sanchez F., Garcia-haro J. (2011). Wireless sensor network deployment for integrating video-surveillance and data-monitoring in precision agriculture over distributed crops. Comput. Electron. Agric..

[38] Ruiz-Garcia L., Lunadei L., Barreiro P., Robla I. (2009). A review of wireless sensor technologies and applications in agriculture and food industry: State of the art and current trends. Sensors.

[39] Pierce F.J., Elliott T.V. (2008). Regional and on-farm wireless sensor networks for agricultural systems in Eastern Washington. Comput. Electron. Agric..

[40] Agudo J.E., Pardo P.J., Sánchez H., Pérez A.L., Suero M.I. (2014). A low-cost real color picker based on arduino. Sensors.

[41] Fisher D.K. (2012). Open-Source Hardware Is a Low-Cost Alternative for Scientific Instrumentation and Research. Mod. Instrum..

[42] Arduino-Home. http://arduino.cc.

[43] Kelley C.D., Krolick A., Brunner L., Burklund A., Kahn D., Ball W.P., Weber-Shirk M. (2014). An Affordable Open-Source Turbidimeter. Sensors.

[44] Koenka I.J., Sáiz J., Hauser P.C. (2014). Instrumentino: An open-source modular Python framework for controlling Arduino based experimental instruments. Comput. Phys. Commun..

[45] Leeuw T., Boss E., Wright D. (2013). *In situ* measurements of phytoplankton fluorescence using low cost electronics. Sensors.

[46] Lian K.Y., Hsiao S.J., Sung W.T. (2013). Intelligent multi-sensor control system based on innovative technology integration *via* ZigBee and Wi-Fi networks. J. Netw. Comput. Appl..

[47] Thalheimer M. (2013). A low-cost electronic tensiometer system for continuous monitoring of soil water potential. J. Agric. Eng..

[48] Tanenbaum J., Williams A. Democratizing technology: Pleasure, utility and expressiveness in DIY and maker practice.

[49] Adafruit Assembled Data Logging shield for Arduino ID: 1141. http://www.adafruit.com/product/1141.

[50] DS1307 64 × 8, Serial, I^2^C Real-Time Clock. http://datasheets.maximintegrated.com/en/ds/ds1307.pdf.

[51] Telefonica. http://www.telefonica.com/en/home/jsp/home.jsp.

[52] Quectel Wireless Solutions-Dedicated M2M Wireless Module Supplier. http://www.quectel.com/.

[53] Robots-Robot-Domestici. http://www.robot-domestici.it/joomla/index.php.

[54] VH400 Soil Moisture Sensor. http://vegetronix.com/Products/VG400/.

[55] Sudha M.N., Valarmathi M.L., Babu A.S. (2011). Energy efficient data transmission in automatic irrigation system using wireless sensor networks. Comput. Electron. Agric..

[56] DS18B20 Programmable Resolution 1-Wire Digital Thermometer. http://datasheets.maximintegrated.com/en/ds/DS18B20.pdf.

[57] EXP TECH. http://www.exp-tech.de/Sensoren/Temperatur/DHT22-temperature-humidity-sensor-extras.html.

[58] MLX90614 Digital, Plug & Play, Infrared Thermometer in a TO-can. http://www.melexis.com/Infrared-Thermometer-Sensors/Infrared-Thermometer-Sensors/MLX90614-615.aspx.

[59] The Comprehensive R Archive Network. http://cran.r-project.org/.

[60] Geesing D., Bachmaier M., Schmidhalter U. (2004). Field calibration of a capacitance soil water probe in heterogeneous fields. Aust. J. Soil Res..

[61] Nonlinear Regression and Nonlinear Least Squares An Appendix to An R Companion to Applied Regression, Second Edition. http://socserv.mcmaster.ca/jfox/Books/Companion/appendix/Appendix-NonlinearRegression.pdf.

[62] Mcmichael B., Lascano R.J. (2003). Laboratory Evaluation of a Commercial Dielectric Soil Water Sensor. Vadose Zone J..

[63] Wang N., Zhang N., Wang M. (2006). Wireless sensors in agriculture and food industry—Recent development and future perspective. Comput. Electron. Agric..

[64] Van Iersel M. (2013). Sensors for improved efficiency of irrigation in greenhouse and nursery production. Hort Technol..

[65] Arduino-ArduinoXbeeShield. http://arduino.cc/en/Main/ArduinoXbeeShield.

